# Improvement of Laser Transmission Welding of Glass with Titanium Alloy by Laser Surface Treatment

**DOI:** 10.3390/ma11102060

**Published:** 2018-10-22

**Authors:** Pin Li, Xingwen Xu, Wensheng Tan, Huixia Liu, Xiao Wang

**Affiliations:** 1 School of Mechanical Engineering, Shanghai Jiaotong University, Shanghai 200000, China; lipsjtu@sjtu.edu.cn or lip@ujs.edu.cn; 2 School of Mechanical Engineering, Jiangsu University, Zhenjiang 212013, China; 18852860868@163.com (X.X.); wx@ujs.edu.cn (X.W.); 3 Changzhou Key Laboratory of Large Plastic Parts Intelligence Manufacturing, Changzhou College of Information Technology, Changzhou 213164, China; tws.163@163.com

**Keywords:** laser transmission welding, laser surface treatment, titanium alloy, high borosilicate glass, surface free energy, elemental diffusion

## Abstract

Laser surface treatment of the titanium alloy was locally oxidized on the metal surface to improve the joint strength of laser transmission welding of high borosilicate glass with titanium alloy. The results find that the welding strength was increased 5 times. The welding mechanism was investigated by the morphology of the welded parts, the tensile-fracture failure mode, the diffusion of the interface elements, and the surface free energy. The results show that there are many adherents between the titanium alloy and high borosilicate glass after tensile fracture, the welding strength was higher when the laser voltage was 460 V, and the tensile–fracture failure mode is mainly ductile fracture. Element-line scanning analysis revealed that elemental diffusion occurred in the two materials, which is an important reason for the high welding strength. Surface free-energy analysis shows that laser surface treatment improves the surface free energy of titanium alloy, promotes the wettability and compatibility, and increases the welding strength of titanium alloy with glass.

## 1. Introduction

Glass to metal seals are widely employed in lighting and electronic devices, automotive, and medical fields [1]. The glass to metal seal is traditional a fusion technique with the glass melted in contact with metal parts to be sealed to. Matched thermal expansion seals, unmatched expansion seals, soldered seals, and mechanical joints are the four major sealing methods of glass to metal [2]. Recently, high-frequency induction heating is used to seal the glass to the Kovar in solar receiver tubes and developed a highly automated process [3]. Due to the nonlinear absorption characteristics of ultrashort-pulsed lasers, ultrashort lasers have attracted significant attention for their application in the fields of cutting [4] and surface mechanical characterization [5]. Laser welding is considered to be a highly-flexible technique with potential for joining glasses and metals. Carter et al. reported systematic analysis and comparison of picosecond laser microwelding of industry relevant Al6082 parts to SiO_2_ and BK7 [6]. Volpe et al. reported on femtosecond laser microwelding of two transparent layers of polymethyl methacrylate (PMMA) based on nonlinear absorption and localized heat accumulation at high repetition rates [7]. For laser transmission welding of glass and metals [8], the laser transmission welding of copper substrates with borosilicate glass was achieved using a femtosecond laser by Itoh et al. Although the melting point and thermal-expansion coefficient of these two materials are quite different, a relatively reliable connection was formed between the two materials [9]. Utsumi reported a direct welding of copper balls with borosilicate glass using a short-pulsed laser [10]. Quintino et al. used a femtosecond laser with a pulse width of 35 fs for laser transmission welding of glass flakes with NiTi alloy flakes. It has been shown that the NiTi particles were formed and splashed in a direction perpendicular to the laser propagation after the laser pulses impacted on the surface. A dimple structure was observed at the weld, indicating a good connection between the two materials [11]. Flury successfully prepared metal lattices on a glass substrate using a glass surface coating to induce reverse transfer by a femtosecond laser [12]. Ciuca et al. used a picosecond laser to achieve transmission welding between quartz glass and aluminum, and found that the nanocrystalline silicon, *γ*-Al_2_O_3_, and *δ*-Al_2_O_3_ were formed in the weld zone [13]. Carter et al. used a picosecond laser to weld a variety of metals such as aluminum, copper, and stainless steel with glass, and found that cracks existed at the interface between metal and glass [14].

The high price of ultrashort-pulsed lasers makes them impractical for application in laser transmission welding technology for welding between metal and glass. Lin et al. used a relatively inexpensive long-pulsed fiber laser to achieve the welding of quartz and anodized aluminum [15]. Wetting is very important to enhance the thermo-mechanical properties in the manufacture of metal matrix composite materials, and reactive infiltration. Narciso et al. enhanced interfacial thermal conductivity in Al/Diamond composites by diamond surface modification [16], and studied the porosity effect on thermal properties of Al-12 wt % Si/Graphite composties [17]. In addition, to improve the strength and hermeticity of the joints, the pre-oxidation was used to form an oxide film on the metal surface. Chern et al. improved the wettability and tightness of 7056 glass on a Kovar surface by pre-oxidizing the surface of Kovar alloy using a furnace thermal treatment [18]. Zhang et al. improved the wettability and diffusivity of borosilicate glass on the surface of Kovar alloy by laser melting oxidation of metal surfaces [19]. Li et al. improved the joint strength of PS and titanium using the two pretreatment methods (laser oxidization treatment and oxygen plasma surface treatment) [20]. Moreover, the laser surface treatment does not require pre-oxidation, and heat preservation of the entire part and the local oxidation of the metal surface can be quickly achieved with good selectivity and repeatability.

The welding of titanium alloy with high borosilicate glass was investigated in this study; it was found that the welding strength of high borosilicate glass with titanium alloy without surface oxidation was very low. To improve the joint strength, the surface of the titanium alloy was first locally oxidized using a semiconductor laser, and transmission welding of titanium alloy with borosilicate glass was then conducted using a long-pulsed Nd:YAG laser. The influence of laser surface treatment on the welding strength of laser transmission welding and the welding mechanism of titanium alloy with high borosilicate glass were studied by analyzing the microstructure, the tensile-fracture failure mode of the weld, the interface elemental diffusion, and the surface free energy.

## 2. Materials and Methods

The main properties of the used materials are shown in Table 1, Table 2 and Table 3. The size of the titanium alloy and the high borosilicate glass samples used in this study was 50 mm × 20 mm × 2 mm. Before beginning the experiment, the samples were cleaned with alcohol in an ultrasonic cleaner and dried in a dry box for 12 h to remove impurities from the material’s surface.

The purpose of laser surface treatment is to form an oxide film on the metal surface. The degree of peroxidation directly influences the strength and hermeticity of the joint. The principle of laser surface treatment is to scan the surface of titanium alloy with a laser under atmospheric conditions, and then the metal is oxygenated by heating to produce a certain thickness of oxide film. A semiconductor laser (Compact 130/140, DILAS Diodenlaser GmbH, Mainz, Germany) was used for laser surface oxidation with an output wavelength of 980 ± 10 nm, a minimum spot diameter of 700–800 μm with a circular shape, a maximum output power of 130 W, and a working temperature kept between 15–25 °C. The process parameters and limits for laser surface treatment are shown in Table 4.

**Figure 1:**
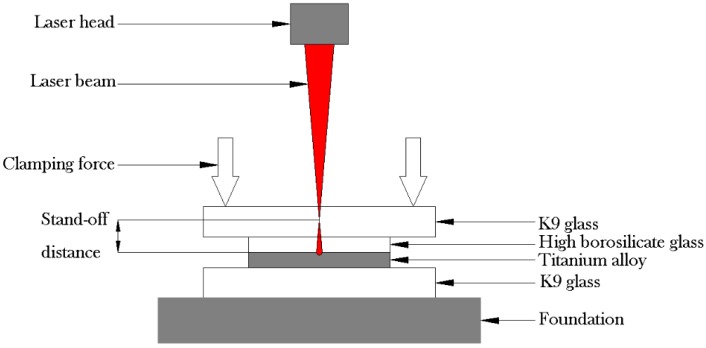
Laser transmission welding schematic.

For the laser transmission welding, a long-pulsed Nd:YAG laser (StarWeld 250, Rofin-Sinar Laser GmbH, Hamburg, Germany) with an output wavelength of 1064 nm was applied. Figure 1 presents a schematic of the principle of laser transmission welding, and the experiment was performed in the form of the lap-joint configuration [21]. The upper-layer material was made of high borosilicate glass with excellent light transmittance, and the lower layer was made of surface-oxidized titanium alloy as the light-absorbing material. In addition, the K9 glass was used as the upper and lower clamping layer and a certain clamping pressure of 0.5 MPa was applied. When the laser was irradiated onto the surface of the titanium alloy transmission through the upper high borosilicate glass, the surface of the titanium alloy absorbed energy, so that the temperature at the interface increased sharply, and makes local melting titanium alloy at high temperature. Under the impact of the pulsed laser, the high-temperature titanium alloy droplets were sprayed around, which caused melting and micro-cracking of the glass surface, forming a small-sized interlocking effect. The welding of the titanium alloy with the glass was thus achieved. The process parameters and limits for laser transmission welding are shown in Table 5. Three replicates were performed for each test condition.

A universal tensile machine (UTM4104, Shenzhen Suns Technology Co., Ltd., Shenzhen, China) was used for testing the joint strength, with v = 2 mm/min. The lap shearing test finally broke the joints through loading tension at both ends of the joints. In this paper, the joint strength is measured by shear stress, and the shear stress is calculated as Formula (1):

(1) $$\sigma =\frac{F}{W\times L}$$

An ultra-depth optical microscopy (VHX-1000, Keyence Corporation, Osaka, Japan) was used to observe the micromorphology of the joint. The interface elemental diffusion analysis was performed using a scanning electron microscope (S-3400N, Hitachi Corporation, Tokyo, Japan).

The contact angle was measured using a contact-angle measurement (OCA40, Dataphysics Instruments GmbH, Stuttgart, Germany). The surface free energy of the material was calculated using the method of Ownes [22]. The surface free energy (*γ*) is taken into account the polar components (*γ^**P**^*) and dispersive components (*γ^**D**^*), and needs to use two test liquids. In this study, pure water and ethylene glycol were used as test liquids. The relevant formulas are shown below [23]:

(2) $$\gamma =\gamma^{D}+\gamma^{P}$$

(3) $$\gamma_{l1}(1+\cos\theta_{1})=2{\left[{\left(\gamma_{l1}^{D}\gamma_{s}^{D}\right)}^{0.5}+\gamma_{l1}^{P}\gamma_{s}^{P}\right)}^{0.5}]$$

(4) $$\gamma_{l2}(1+\cos\theta_{2})=2{\left[{\left(\gamma_{l2}^{D}\gamma_{s}^{D}\right)}^{0.5}+\gamma_{l2}^{P}\gamma_{s}^{P}\right)}^{0.5}]$$

where $\gamma_{l1}$ is the surface tension of pure water ($\gamma_{l1}$ = 75 mN/m); $\gamma_{l2}$ is the surface tension of ethylene glycol ($\gamma_{l2}$ = 48 mN/m); $\theta_{1}$ represents the surface contact angle of pure water; $\theta_{2}$ represents the surface contact angle of ethylene glycol; $\gamma_{l1}^{D}$ and $\gamma_{l2}^{D}$ are the dispersive components of the surface tension of pure water and ethylene glycol, respectively ($\gamma_{l1}^{D}$ = 21.6 mN/m, $\gamma_{l2}^{D}$ = 29 mN/m); and $\gamma_{l1}^{P}$ and $\gamma_{l2}^{P}$ represent the polar components of the surface tension of pure water and ethylene glycol, respectively ($\gamma_{l1}^{P}$ = 53.4 mN/m, $\gamma_{l2}^{P}$ = 19 mN/m).

## 3. Results and Discussion

### 3.1. Effect of Laser Surface Treatment on Welding Strength

**Figure 2:**
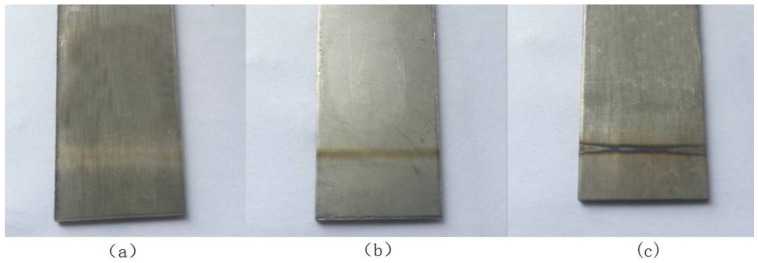
Surface morphology of titanium alloy after treatment by surface treatment power (**a**) 40, (**b**) 50, and (**c**) 60 W.

Figure 2 shows the surface morphology of the titanium alloy after laser oxidation treatment with a spot diameter of 2 mm, a scanning speed of 5 mm/s, a number of scans of 8, and laser surface treatment powers of 40, 50, and 60 W. When the laser surface treatment power was 50 W, the surface treatment was achieved with no obvious occurrence of melt, and the oxide layer was also formed.

**Figure 3:**
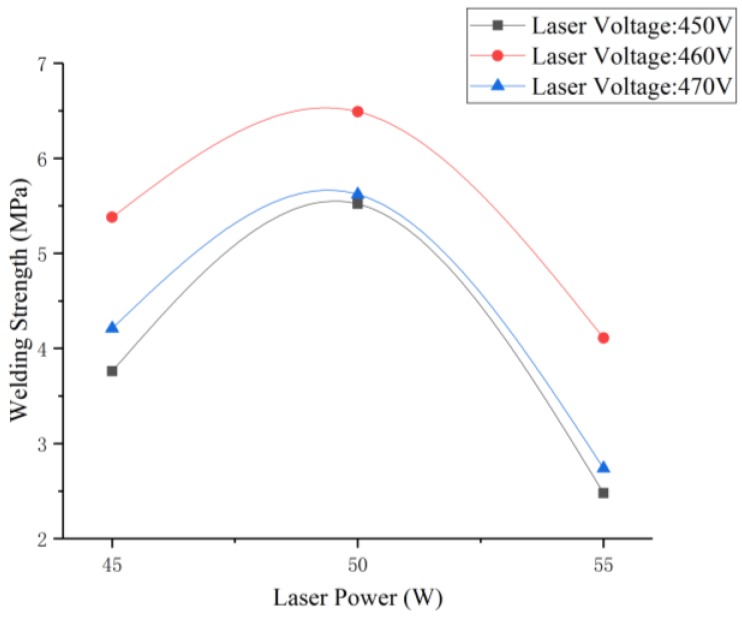
Effect of laser surface treatment power on welding strength.

The laser transmission welding of titanium alloy without laser surface treatment with glass was carried out first. It was found that the welding strength was only 1.31 MPa when the welding speed was 3 mm/s with a laser voltage of 460 V, a laser frequency of 10 Hz, a laser pulse width of 2.5 ms, and a defocusing amount of 0 mm. Figure 3 shows the effect of laser surface treatment power (45, 50, and 55 W) on welding strength.

**Figure 4:**
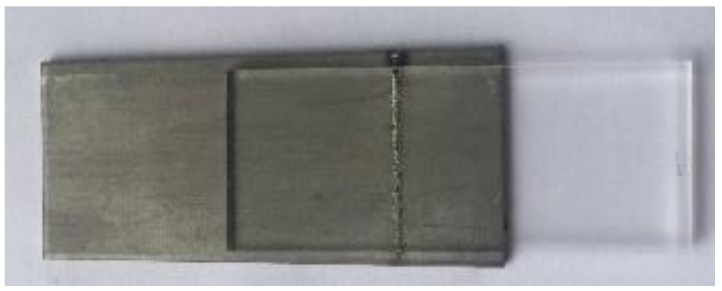
Welding between titanium alloy and high borosilicate glass.

As shown in the Figure 4, when the laser surface treatment power was 50 W, the laser voltage for transmission welding was 460 V, the welding speed was 3 mm/s, the laser frequency was 10 Hz, the laser pulse width was 2.5 ms, and the defocusing amount was 0 mm, the welding strength was the highest (6.49 MPa), which was 5 times that of the welding strength without laser surface treatment. Figure 4 shows the sample of laser transmission welding between titanium alloy and high borosilicate glass. It was found that the oxide film formed on the surface of the titanium alloy had a tendency to adhere to the high borosilicate glass [24], which is favorable for the improvement of the laser transmission welding strength.

### 3.2. Micromorphology of Welding Seam and Tensile Failure Analysis

**Figure 5:**
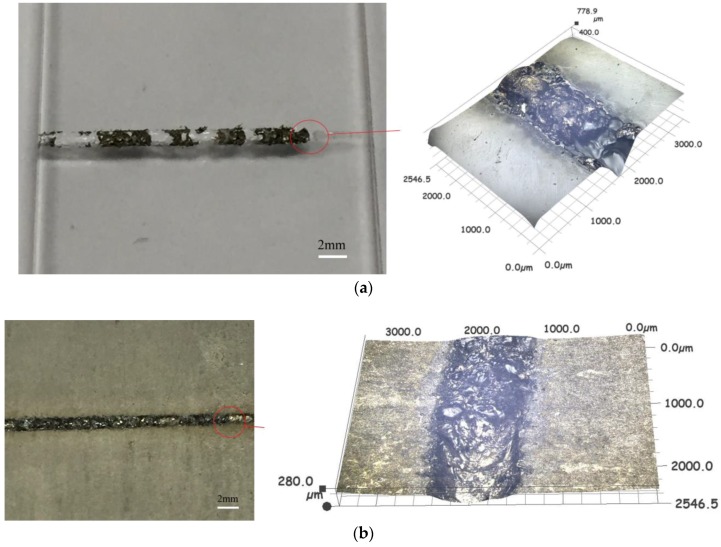
Micrographs of titanium alloy and high-borate borosilicate glass welds after tensile test: (**a**) glass and (**b**) titanium alloy.

An ultra-depth optical microscopy was used to observe the micromorphology of welding seam after tensile test. Figure 5 shows the micrographs of titanium alloy and high borosilicate glass welds after tensile test with 100×. Under high temperature, the laser treated titanium alloy chemically reacted with SiO_2_ in the glass and formed black adherents. These black adherents differed from the initial glass form and were relatively firmly attached to the glass, forming a small-size interlock effect.

**Figure 6:**
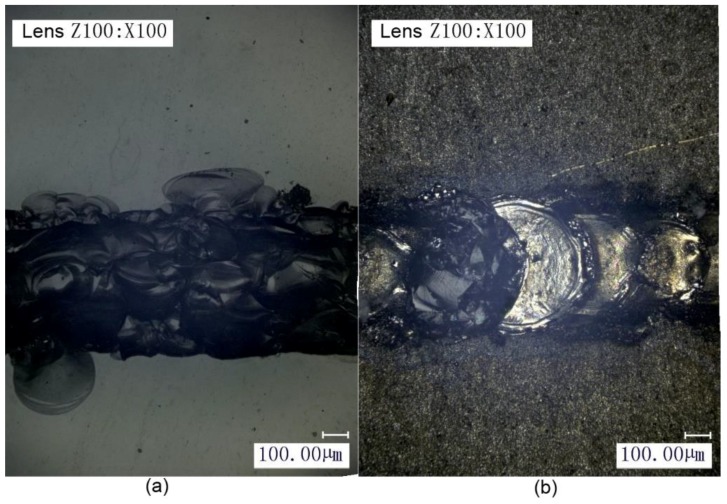
Micrographs of titanium alloy and high borosilicate glass using laser voltage for transmission welding of 440 V: (**a**) glass and (**b**) titanium alloy.

**Figure 7:**
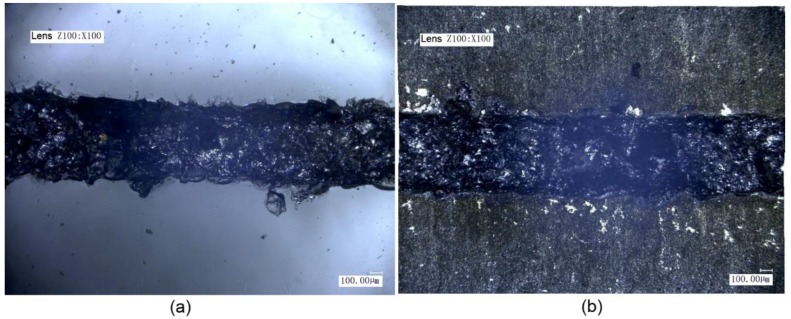
Micrographs of titanium alloy and high borosilicate glass using laser voltage for transmission welding of 460 V: (**a**) glass and (**b**) titanium alloy.

**Figure 8:**
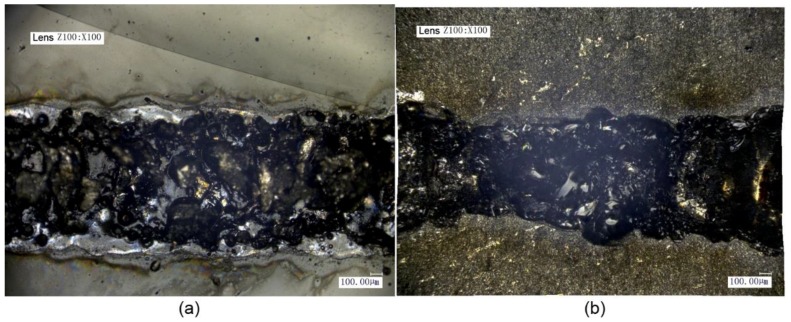
Micrographs of titanium alloy and high borosilicate glass using laser voltage for transmission welding of 480 V: (**a**) glass and (**b**) titanium alloy.

When the laser surface treatment power was 50 W, the welding speed was 3 mm/s, the laser frequency was 10 Hz, the pulse width was 2.5 ms, the defocusing amount was 0 mm, and the laser voltages for transmission welding were 440, 460, and 480 V; the welding strengths obtained were 5.52, 6.49, and 5.62 MPa, respectively. The morphologies of the welds after tensile fracture are presented in Figure 6, Figure 7 and Figure 8.

It can be seen from Figure 6 that when the laser voltage for transmission welding was 440 V, no adherent was formed at the welding seam. This is because the laser voltage was small and the energy input was insufficient. Although droplets were splashed on the surface of the titanium alloy, they did not have a sufficient chemical reaction with the glass. A row of concave structures were only formed on the surface of the titanium alloy, resulting in un-ideal welding.

**Figure 9:**
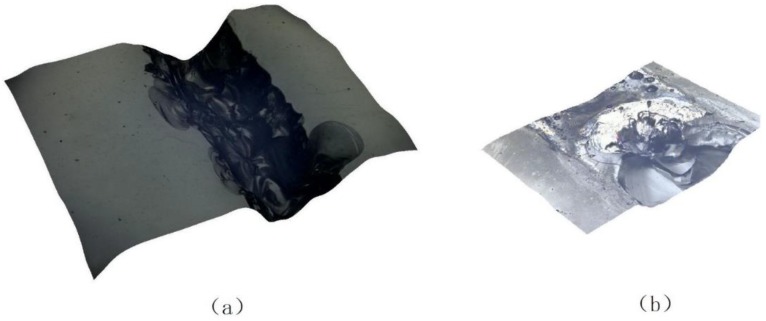
Micrographs of the weld at 500×: (**a**) glass and (**b**) titanium alloy.

The welding seam of the titanium alloy and the glass was uniform and aesthetic when the laser voltage for transmission welding was 460 V, as shown in Figure 7. When magnified 500×, a dimple structure at the welding interface between the titanium alloy and the glass was observed [25] and indicating that a good weld existed between the titanium alloy and the glass, as shown in Figure 9.

It can be seen from Figure 8 that when the laser voltage for transmission welding was 480 V, there were slight breakages and obvious cracks at the glass weld, and ablation occurred at the weld of the titanium alloy. The heat and impact generated by the pulsed laser resulted in a brittle failure on the glass, which seriously affects the weld and the welding strength.

**Figure 10:**
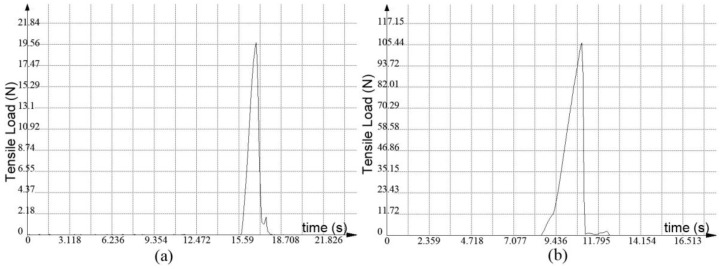
Tensile load changes with time at laser voltages for transmission welding of (**a**) 440 and (**b**) 460 V.

Figure 10 shows the changes of tensile load as a function of time in a tensile test recorded by a UTM4104 universal testing machine. It can be seen from Figure 10a that when the laser voltage for transmission was 440 V, uniform relative movement occurred to the titanium alloy and glass with the testing machine, which had a uniform linear motion, until the breaking force reached the maximum value, at which time the relative displacements of the titanium alloy and glass were small. Since the fracture of the weld is flush and bright, and perpendicular to the direction of the normal stress, the fracture failure mode is mainly brittle fracture.

It can be seen from Figure 10b that when the laser voltage for transmission welding was 460 V, the maximum value of the breaking force was reached after a certain displacement between the titanium alloy and the glass was formed, when they moved with the testing machine, which has a uniform linear motion. The adherents after fracture were observed at the weld fracture, which indicates that a good weld was achieved between the titanium alloy and the glass, and the failure form is mainly ductile fracture.

### 3.3. Diffusion of Elements at the Joint Interface

**Figure 11:**
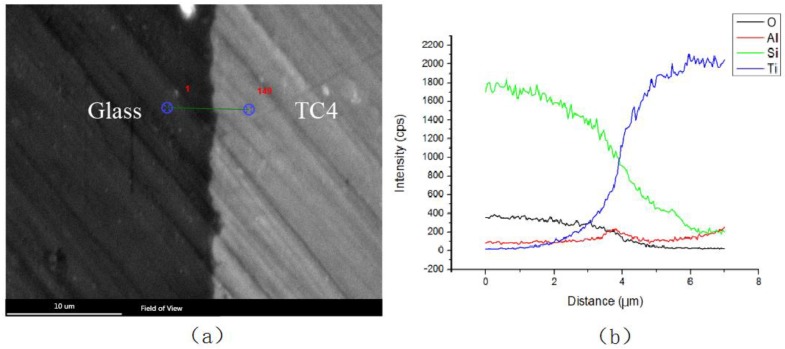
Scanning-electron-microscope (**a**) micrographs of cross-section and (**b**) element distribution along scan line.

In order to further explore the mechanism for the formation of welded joints between titanium alloy with high-borate borosilicate glass during laser transmission welding, the cross-section morphology of the sample was observed by an S-3400N scanning electron microscope. The joint interface of the weld was investigated with an X-ray energy spectrometer for elemental-line scanning analysis to obtain the chemical composition in the weld (Figure 11).

The green and blue lines in Figure 11 represent the changes in the content of Si and Ti, respectively. There was a weak inter-diffusion between elemental Si and Ti at the joint interface. The thickness of the diffusion transition layer was approximately 4 μm. Under laser pulses impact, obvious elemental diffusion phenomenon occurred between the glass and titanium alloy, which is an important reason for the formation of high-strength welded joints.

### 3.4. Contact Angle and Surface Energy

The surface contact angle is the angle between the tangent of the droplet profile and the solid surface it contacts, and it is an important indicator of the wettability of solid surfaces [26]. If *θ* < 90°, the solid surface is hydrophilic, and the smaller the contact angle, the better the wettability. If *θ* > 90°, the solid surface is hydrophobic, and the liquid does not wet the solid easily, but will move on the surface easily.

**Figure 12:**
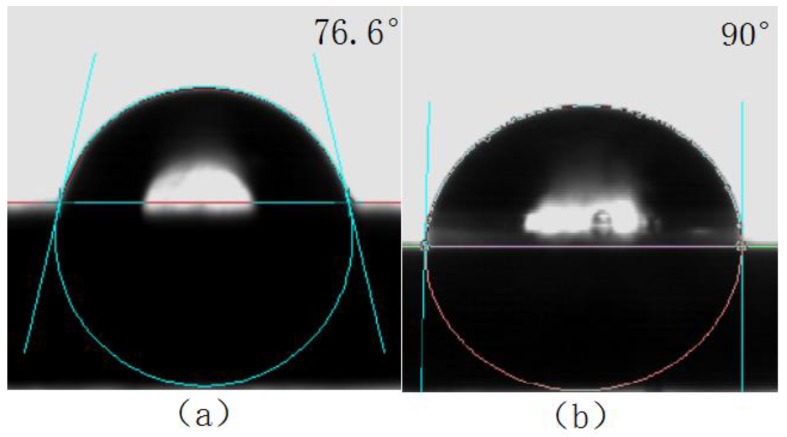
Water contact angle of titanium alloy (**a**) with an (**b**) without laser treatment.

**Figure 13:**
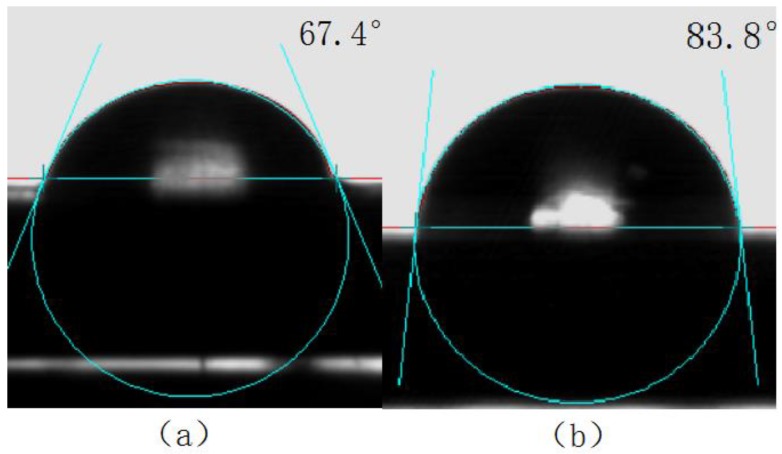
Ethylene glycol contact angle of titanium alloy (**a**) with and (**b**) without laser treatment.

The surface contact angles of the titanium alloy with and without laser surface treatment were separately measured by a CA040 surface contact angle measurement, and are shown in Figure 12 and Figure 13, respectively.

As can be seen from Figure 12 and Figure 13, the surface contact angle of the titanium alloy after laser surface treatment decreased and the wettability improved. The surface free energy of the titanium alloy before and after laser surface treatment was calculated using Equations (3) and (4); the results are shown in Table 6. The surface energy of the titanium alloy increased after laser surface treatment, which promoted the compatibility between the titanium alloy and the glass, thus improving the welding strength during laser transmission welding.

Other researchers have also shown that the surface free energy of materials is an important factor affecting the welding strength when they were studying laser transmission welding. For example, Liu et al. investigated the laser transmission welding of PA66 with PVC [27]. It was found that the surface free energy of PA66 after surface magnetron sputtering of a layer of Al increased, so that PA66 and PVC, which could not be welded originally, were welded, forming a reliable connector through laser transmission welding by using Al atoms as a transition layer. This indicates that the increase of the material’s surface free energy is beneficial to improving the strength of the joint.

## 4. Conclusions

The laser transmission welding of high borosilicate glass with titanium alloy was conducted. It was found that the welding strength could be improved by laser surface treatment of the titanium alloy. The joining mechanism was studied by the morphology, tensile-fracture mode, the elements diffusion, the surface free energy, and the following conclusions were drawn.

(1) A large number of black adherents were observed at the weld between the laser-surface-treated titanium alloy and the high borosilicate glass. A small-size interlock effect may be formed, resulting in a strong joining effect;
(2) The welding strength was the higher when the laser voltage for transmission welding was 460 V, and the tensile-fracture failure mode was mainly ductile fracture. When the voltage for transmission welding was high, the titanium alloy was ablated due to excessive energy input, and the glass was cracked, thus reducing the welding strength;
(3) It was found that there was elemental diffusion at the weld interface between the titanium alloy and the glass, which is an important reason for the formation of high-strength welded joints;
(4) The measurement of the surface contact angles of the titanium alloy shows that the laser surface treatment of the titanium alloy increased the surface energy and promoted the compatibility between the titanium alloy and the glass, thus improving the welding strength of the two materials.

## Figures and Tables

**Table 1:** Main properties of titanium alloy and high borosilicate glass.

Property	Titanium Alloy	High Borosilicate Glass
Density (g/cm^3^)	4.5	2.23
Specific heat J/(kg K)	520	98
Thermal conductivity (W/mK)	7.95	1.2
Melting temperature, Tm (°C)	1660	1680
Ultimate tensile stress (MPa)	895	40–100

**Table 2:** Content of elements in TC4 titanium alloy.

Chemical Composition	Mass Percentag
Ti	Bal
Fe	≤ 0.3%
C	≤ 0.1%
N	≤ 0.05%
O	≤ 0.015%
H	≤ 0.2%
V	5.5~6.8%
Al	3.5~4.5%

**Table 3:** Compositions of high borosilicate glass 3.3.

Chemical Composition	Mass Percentag
SiO_2_	80.4%
B_2_O_3_	12.7%
Al_2_O_3_	2.4%
Na_2_O & K_2_O	4.2%
Others	0.3%

**Table 4:** Process parameters and limits for laser surface treatment.

Parameter	Limits		
Laser power for surface treatment (W)	40	50	60
Laser scanning speed (mm/s)	5	5	5
Number of scans (n)	8	8	8
Spot diameter (mm)	2	2	2

**Table 5:** Process parameters and limits for laser transmission welding.

Parameter	Limits			
Laser voltage for transmission welding (W)	450	460	470	480
Laser welding speed (mm/s)	3	3	3	3
Laser frequency (Hz)	10	10	10	10
Laser pulse width (ms)	2.5	2.5	2.5	2.5
Stand-off distance (mm)	0	0	0	0

**Table 6:** Surface free energies of titanium alloy.

Material Type	*γ* (mN/m)	*γ^D^* (mN/m)	*γ^P^* (mN/m)
Titanium alloy	19.31	16.45	2.86
Laser-treated	23.60	22.01	1.6
